# The role of optometry in healthcare for visually impaired older adult populations: a Swiss case study

**DOI:** 10.3389/frhs.2024.1378236

**Published:** 2024-11-25

**Authors:** Alexander Seifert, Daniela S. Nosch

**Affiliations:** ^1^ School of Social Work, University of Applied Sciences and Arts Northwestern Switzerland (FHNW), Olten, Switzerland; ^2^ Institute of Optometry, University of Applied Sciences and Arts Northwestern Switzerland (FHNW), Olten, Switzerland

**Keywords:** older adults, visual impairment, optometry, healthcare, consulting services

## Abstract

**Background:**

Visual impairment (VI) is common among older adults aged 70 years and older, and its prevalence increases with advancing age. The optometry profession may play an important role in a patient-centred health system that incorporates medical and psychosocial aspects by working closely with low vision counselling services (LVCS). This paper investigates the current level of cooperation between optometry and LVCS by analysing the referral practice of optometrists to LVCS for the older population with VI, based on the PROVIAGE study.

**Methods:**

A national, telephone-based survey of individuals aged ≥70 years and an online survey of professionals in ophthalmology, optometry and LVCS was conducted in 2022 across Switzerland.

**Results:**

The responses of 154 individuals with VI and 272 professionals (123 ophthalmologists, 126 optometrists and 23 staff of low vision rehabilitation consulting centres) were analysed. Among the respondents with age-related VI, 33.1% stated that they were aware of LVCS. Of these, however, only 11.7% reported that they had visited such centres during the last five years. Sixty-eight percent of respondents attended the ophthalmologist, but only 1.3% went to the optometrist for vision-related problems. Among ophthalmologists, 95.9% indicated that they had referred patients to LVCS, whereas only 58.8% of optometrists had done so.

**Conclusions:**

The results of this study highlighted the relationship between the different clinician referrals, patient needs, and potential barriers preventing referrals towards older adults in Switzerland. A stronger cooperation between professions in the care network will enhance vision care for the ageing population without the need for additional infrastructure.

## 1 Introduction

Visual impairment (VI) is prevalent among older adults aged 70 years and older, and its prevalence increases with advancing age (1, 2). As demographic changes progress (3), the population of older individuals experiencing age-related vision loss and impairment is expected to continue to grow in upcoming years. The current global estimate projects that the number of individuals with moderate to severe VI will surge to 588 million by 2050 (4). This projected boom in the size of this cohort may be fuelled by pathologies typically pervasive among ageing populations, such as cataracts and/or age-related changes to the retina (e.g., age-related macular degeneration and diabetic retinopathy), the neural pathways (e.g., glaucoma) and the cortex. Non-pathological age-related deficits that critically impact day-to-day activities, include deteriorating spatial contrast sensitivity, scotopic function and visual processing speed (5).

Advancing age is commonly viewed as a nonpreventable cause of VI among ageing adults. Therefore, many individuals in ageing populations – especially those in care homes or those receiving care at home – accept deterioration in their vision as a *normal* aspect of the ageing process and do not undergo or request regular eye examinations (2). However, there is extensive literature on the psychosocial and functionality challenges faced by older adults experiencing age-related VI (6, 7). Therefore, the individual coping of older adults with the demands of daily life in older age (e.g., coping with the loss of family members), while at the same time coping with a disability that first appears around the age of 70 due to sensory impairment, is a general challenge for medical-oriented health care systems, where the psychosocial needs of older adults are not primarily met. In other words, the psychosocial component of coping with daily life in later life (especially for those who experience a disability for the first time at an advanced age) needs to be considered in health care for ageing adults, in addition to medical clarification. This is certainly not something that can be delivered in ophthalmology treatment sessions. It requires instead socially oriented specialists, who are for example operating in dedicated Low Vision Counselling Services (LVCS) (8, 9).

Hence, it is imperative to raise awareness of the necessity of undergoing regular eye examinations and utilising low vision rehabilitation services among members of the (para)medical professions who care for individuals of advanced age and non-professional individuals in their personal environment. Because ageing adults represent the fastest-growing segment of the human population and because increasing longevity is not linked to a life free of disabilities, there will soon be a growing demand for eye treatment, low vision rehabilitation and, most especially, consulting services from VI consultants who focus on psychosocial concerns that are not addressed in the medical consultation setting.

In addition to treatment by ophthalmologists, which is an important component of eye-specific primary care, optometry professionals delivering eye care are ideally positioned to act as *gatekeepers* to provide primary eye care for older adult populations with VI. Optometric professionals are easily accessible, scheduled appointments are available without waiting times, and they may offer mobile services – delivered at an individual's home or care home. Based on findings from an eye examination, they may offer consultation, provide visual aids (e.g., magnifying lenses and spectacles) and/or may refer individuals to an ophthalmologist for further medical treatment and/or to social services for low vision services. In addition, optometrists working at an optician's practice can offer low vision consultations and refer for psychosocial issues to the appropriate counselling centres, employing a person-centred approach to individual care management.

However, although optometry beside ophthalmologists – with respect to visually impaired older adults – has the potential for a patient-centred health system that incorporates medical and psychosocial aspects, little is known about the extent to which older individuals perceive optometrists (beside ophthalmologists) as essential professionals in the healthcare system or the extent to which optometrists themselves handle counselling on psychosocial issues in their day-to-day practice. This raises the following question: *What is the current situation regarding referral practices among optometry professionals with respect to social consulting services for older visually impaired people, and to what extent can optometry play a pivotal role here?*

Based on the *PROVIAGE* (“Professional network for visual impairment in old age”) study, which investigated the cooperation between medical and rehabilitative care (social consulting services) professionals who offer their services to older individuals in Switzerland, we report the study's descriptive findings on the involvement of the optometric profession to answer the aforementioned research question.

## 2 Methods

The *PROVIAGE* study addressed the question of what referral gaps exist between medical and non-medical specialists in Switzerland who care for older individuals experiencing VI (10). Schweizerischer Zentralverein für das Blindenwesen (SZBLIND) and Retina Suisse commissioned the School of Social Work at the University of Applied Sciences Northwestern Switzerland (FHNW) for this study. This study included the following: (a) a national telephone-based survey of individuals aged ≥70 years and (b) an online survey of professionals in ophthalmology, optometry and LVCS. The national telephone and online surveys were conducted from October to November 2022 across Switzerland – in German, French and Italian.

### 2.1 Telephone survey of individuals aged ≥70

A random sample of the permanent resident population of Switzerland aged ≥70 years were selected from the AZ-Direct database, which is based on a public phonebook. A total of 1,611 adults aged ≥70 years from the main language regions of Switzerland (German, French and Italian) were finally interviewed, using a computer-assisted telephone interview format. The participants were asked to state their age, gender and whether they had a VI that was acquired later in life [For example: “Is your visual impairment related to a specific eye disease?”: Which in most cases was age-related macular degeneration (46.6%), cataracts (28.2%) and glaucoma (16.5%)]. They also rated their ability to cope with day-to-day life on a scale of 1‒10 (1 = very poor and 10 = very good). Additional questions focused on the following: their general situation in life; how often they underwent medical checkups; who they turn to for help concerning general and medical challenges; and, more specifically, whether they used LVCS – whether these services were recommended to them, and if so, by whom; and finally, whether they were content with the service they had received. A total of 154 individuals fulfilled the requirements for the target population of older adults with VI.

### 2.2 Online survey of professionals

Ophthalmologists, optometrists and managers of LVCS were surveyed using a standardised online questionnaire. A random sample of addresses of professionals available online was used to generate the sample population for this survey. The respondents were asked the following: to evaluate the importance of LVCS on a five-point scale (1 = not important at all, 2 = rather not important, 3 = partly important, 4 = rather important, 5 = very important); whether they had received any related (continuing) education; about their interactions with other related professions regarding VI; to mention the specific LVCS they had been in contact with; and to provide information about their referral practice (i.e., referral frequency, who they refer patients to, criteria for a referral and existing hurdles).

### 2.3 Ethical considerations

Ethical review and approval were not required for the study on human participants via telephone survey in accordance with the local legislation and institutional requirements. The internal ethics committee of the School of Social Work at the University of Applied Sciences Northwestern Switzerland (FHNW) approved the study. The study complies with the Declaration of Helsinki. Participants received detailed information prior to the telephone survey, and they consented verbally. They were made aware that they could withdraw their consent to participate in the telephone survey at any time during the interview. All identifiable personal data were anonymised and the keycodes were deleted after completion of the study.

## 3 Results

For the national telephone-based survey, the 1,611 individuals aged ≥70 years, who were interviewed via telephone, were distributed across Switzerland as follows: 1,024 (64.4%) in the German-speaking part, 394 (24.8%) in the French-speaking part and 173 (10.9%) in the Italian-speaking part. Females accounted for 64.9% of the respondents, 254 individuals were older than 84 years, the mean age was 78 years, the youngest respondent was 70 and the oldest was 94 years old. In the 70–79 age group, 65.5% were female and 34.5% were male; in the 80–89 age group, 63.9% were female and 36.1% were male; in the 90–94 age group, 66.7% were female and 33.3% were male. One hundred and fifty-four individuals indicated having age-related VI. The ability to cope with day-to-day life independently, was rated higher [*t* (1,537) = 4.6, *p* < 0.001] among individuals with no VI (M = 9.04 ± 1.41) than among those who stated that they were affected by a VI acquired later in life (M = 8.47 ± 1.69).

Regarding the online survey of professionals, a total of 272 professionals replied: 123 ophthalmologists (47% of whom were practising at their own private ophthalmology practice), 126 optometrists and 23 managers of LVCSs. For the analysis presented in this study, we focus on the optometrists (furthermore, in comparison to the ophthalmologists). Among the optometrists, 37.3% were female, 62.7% were male, the age range was 22–80 years (mean age = 49.8), and 22 were retired optometrists.

### 3.1 Who do older adults turn to for help and which professionals refer older adults to LVCS?

The following question captures a central issue in this study: When older adults with VI require advice regarding their vision problems, who do they turn to first? The results of the telephone-based survey of individuals show that ophthalmologists are most often approached first (68%), and only afterwards do they turn to family members. Much less frequently the family doctor is also consulted (7.2%). Other persons or LVCSs for the visually impaired were contacted even less frequently; only 1.3% of the respondents mentioned consulting an optician.

Among the respondents with age-related VI, 51 (33.1%) stated that they were aware of LVCS. Of these however, only 18 (11.7%) indicated that they had consulted a LVCS during the last five years. Those, having consulted them, had only done so once or twice during the last five years. In half of these cases, they were referred to the centres by their ophthalmologist – in the other half they were referred by family members, friends, and other sources (such as an optometrist). The reason for this is that optometrists refer their patients to ophthalmologists and not directly to low vision rehabilitation centres.

Of the 136 respondents with age-related VI, who had not yet visited a LVCS, only 16 (11.8%) indicated that they had received a referral to one; of these, only two were referred by their ophthalmologist. In most cases, it was family members and friends, who mentioned LVCS. Sometimes, it was also an optometrist or a general practitioner. This indicates that there is considerable potential for optometrists to increase their referral rates to such rehabilitation centres. On examining the impact of LVCS’ on individuals’ self-assessment of their life situation, a statistically significant but modest Spearman two-side-correlation (*r*_s_ = 0.143, *p* = .041) was observed between utilising the LVCS (yes/no) and individuals’ subjective evaluation of their ability to cope with day-to-day life (“ability to cope with everyday life independently”; see Method section for scale). This indicates that individuals who have sought guidance from LVCS tend to express a slightly more positive outlook on their ability to independently manage daily life compared to those who have not accessed such services.

### 3.2 How do optometrists rate the importance of visiting a centre for the visually impaired?

All the optometrists surveyed indicated that they are consistently serving an increasingly larger proportion of older adults. This raises the question of whether optometrists also consider the psychosocial aspects of eye care to be important. The answers to this question reveal that all the professionals surveyed view it as important to advise older visually impaired adults on practical life, psychological and social issues in addition to the need for medical clarification. On a scale of 1 (“very unimportant”) to 5 (“very important”), the average score among the ophthalmology professionals was 4.42 ± 1.26; for optometry professionals, it was 4.37 ± 1.14, and for managers of LVCS, it was 4.52 ± 1.20. These scores indicate that optometrists rate this aspect slightly lower than members of the other professions. In general however, optometrists also rate this aspect highly, showing that they consider not only the functionality aspects of vision but also the psychosocial component when caring for their patients. In addition, experience with the psychosocial aspects of eye care during undergraduate training of optometrists is crucial for acknowledging the importance of these non-medical aspects of coping with everyday life for older adults; Nevertheless, only 50.0% of the optometrists indicated that they received inputs about this during their study.

### 3.3 What interactions do optometrists have with LVCS and other specialists?

In addition to the theoretical knowledge available via (continuing) education, it is also critical to have a personal experience of the services offered by LVCS. However, only 39.2% of the respondents of the online survey indicated that they themselves had visited a LVCS before. This was 48.8% of the ophthalmologists surveyed and 36.5% of the optometrists.

Furthermore, we explored whether and to what degree specialists in different professions interact professionally with each other. The surveyed ophthalmologists indicated that they have the most substantial interactions with optometrists (Table 1), and in some instances, there is also cooperation with LVCS. Although the exchange of patient-related information notably enhances interactions with other specialist professionals, it is evident that these interactions are not consistently intensive and vary significantly based on individual circumstances.

**Table 1:** Interaction between three specialist professions.

		Interactions with		
		Ophthalmologists	Optometrists	LVCS
Specialist professionals	Ophthalmologists	–	3.10	2.85
	Optometrists	3.40	–	2.24
	LVCS	3.90	3.62	–

Scale: 1 = “no contact” to 5 = “very intensive (very good interaction)”.

Optometrists primarily communicate with ophthalmologists, while their interaction with LVCS is notably less frequent. Interestingly, these LVCS exhibit a rather intensive level of interaction with optometrists. In principle, there is room for enhanced communication exchanges between optometrists and LVCS. Both, optometrists and LVCS reported the least frequent interactions despite addressing similar social issues regarding older individuals, such as giving advice concerning technical aids and strategies for coping with aspects of day-to-day life related to vision.

The online survey questionnaire also inquired about the depth of knowledge that members of each specialist profession possessed regarding the services and advisory offerings provided by LVCS. While over half (57.7%) of the ophthalmologists responded affirmatively, selecting the “*rather yes*” response, the corresponding figure for the optometrists was only 29.2%. Among the optometrists, a substantial 62.0% admitted to having limited or no information about the services offered by LVCS.

### 3.4 Referral practice

When considering all the professionals surveyed, a substantial 72.8% had referred one or more older individuals to a LVCS during the last twelve months. Among the ophthalmologists alone, the figure was 95.9%, and it was 58.8% among the optometrists. On average, the professionals who had issued referrals over the last twelve months, gave referrals to eight individuals each, with approximately seven of the referred individuals actively utilising the services. This is reflected in the high positive correlation (*r* = 0.946, *p* < 0.001) between referral and active usage of the service. However, it is noteworthy that some referred individuals do not ultimately avail themselves of the recommended service.

Referral practice also exhibits a variation among the groups of specialist professionals, with ophthalmologists reporting referrals more frequently than optometrists. Nevertheless, the success rate of older individuals seeking advice from the centres to which they were referred, was significantly high for referrals from both, optometrists and ophthalmologists (Table 2).

**Table 2:** Referral practice.

		Ophthalmologists	Optometrists
Mean values (number of persons)	Transferred in the last twelve months	11	4
	Have used the advisory service	9	4
Significance test (linear binary regression between numbers of referral and utilisation, grouped per specialty professions)		Beta = 0.945, *p* < .001	Beta = 0.930, *p* < .001

Among optometrists, who referred patients, the prevailing sentiment was positive, with a significant majority indicating that such referrals had a beneficial impact. Specifically, 23.8% acknowledged that referrals were immensely helpful, while a substantial 66.7% rated the benefit of referrals as “*rather yes*”. Only a modest 9.5% responded in the negative when asked about the perceived effectiveness of patient referrals.

The online survey questionnaire also inquired about the perceived responsibility for initiating a referral to a LVCS or making a relevant recommendation. Ophthalmologists emerged as the professionals most identified as the first point of contact for referrals and recommendations, while patients and their relatives were mentioned less frequently. Nonetheless, each group of specialist professionals acknowledged a certain level of responsibility in this regard. Notably, a significant 83.3% of optometrists consider themselves responsible for encouraging older adult patients to seek guidance from a LVCS.

### 3.5 Perceived hurdles and the expectations of LVCS

In addition to the question of responsibility, the question of the potential hurdles associated with referrals was raised. Among optometrists, the most common reason given (40.5%) was that “*the older people concerned feel put off by advice centres for the blind and disabled*”. This was followed by “*I have no information about local*” LVCS (31.0%). Other reasons were indicated much less frequently (Table 3). Nevertheless, none of the respondents selected the response “*I don't see any benefit in it*”, indicating that optometrists perceive a general usefulness in the work done by the LVCS. Ophthalmologists have a slightly different ranking of the barriers they face, for example they rate “I don't have enough time for that” higher (23.6%) than optometrists (4.8%).

**Table 3:** Referral hurdles.

Percentages (multiple answers possible)	Optometrists	Ophthalmologists
The older people concerned feel put off by LVCS for the *blind* and *disabled*.	40.5	29.3
I have no information about LVCS.	31.0	11.4
I have no contact with LVCS.	24.6	6.5
The older people concerned are not open to this.	21.4	28.5
There are no LVCS here in my area.	19.8	4.9
Affected persons cannot afford the counselling financially.	14.3	11.4
I don't have enough time for that.	4.8	23.6
It is not my job to make the referral (recommendation).	3.2	0.0
I don't see any benefit in it.	0.0	0.0

The professionals were also queried about their expectations from a consultation at a low vision rehabilitation centre (Table 4). Optometrists anticipate that their patients will receive support on practical life and psychosocial issues. Their expectations centred very little around receiving some form of relief regarding their workload. Instead, LVCS are viewed as a complementary resource to medical therapy. Ophthalmologists show a similar ranking of expectations, although they rank “helping patients with practical matters” slightly higher as the most important expectation.

**Table 4:** Expectations regarding what LVCS should provide.

Percentages (multiple answers possible)	Optometrists	Ophthalmologists
Help for patients with social and psychosocial issues	77.0	87.8
Help patients with practical life matters	76.2	91.1
Support medical therapy	27.8	23.6
Professional relief for me	6.3	22.0

## 4 Discussion

The national telephone survey and the online survey conducted in this study were aimed at investigating the level of cooperation between medical and rehabilitative care (social consulting services) professionals who care for older adults in Switzerland, with a focus on the optometric profession. The referenced PROVIAGE study is the first national study in this research field to investigate referral practices in Switzerland – and, to the best of our knowledge, globally. Based on the data from the national telephone survey, older adults with VI are most likely to turn first to their ophthalmologist for help before consulting a general practitioner or family member. They very rarely turn to their optometrists first when they have questions regarding their vision. This emphasises the potential for expanding the role of the optometric profession in primary eye care and, ultimately, the need to increase the rate of referrals to LVCS. Considering the easy accessibility that typifies optometrists, leveraging their involvement in advising patients could prove highly valuable. This approach would ensure that the responsibility of informing patients about the existence of LVCS does not fall solely on ophthalmology specialists. Furthermore, establishing collaboration with LVCS would complement the existing services provided to patients by optometrists.

The importance of advising older adults with VI to seek counselling on practical life, psychological and social issues (11, 12) – in addition to medical treatment – was rated highly by all the professionals, who participated in the online survey. Although optometrists communicate frequently with ophthalmologists, they interact much less with LVCS. This highlights the potential for the optometric profession to increase the scope of its practice in the coming years, which would ultimately contribute to a better utilisation of the already existing infrastructure. Nevertheless, the findings of this study also show that there are hurdles associated with potential referrals. Among optometrists, the most common hurdle indicated was that “*the older people concerned feel put off by advice centres for the blind and disabled*”. This was followed by “*I have no information about local*” LVCS (31.0%). These responses make it clear that, on the one hand, optometrists believe that older adults, who develop sight problems for the first time in their older age, feel put off by the labelling “counselling centres for the blind” or “counselling centres for the disabled” applied to LVCS. On the other hand, there is also a very practical information gap in the form of a lack of knowledge about the current services offered by local LVCS. This is where information campaigns about the services provided by LVCS and closer cooperation between optometrists and LVCS can be of immense benefit. Nonetheless, none of the respondents selected the “*I don’t see any benefit in it*” response as a referral hurdle, indicating that optometrists perceive a general usefulness in the service provided by LVCS, particularly when it supports the day-to-day work of optometrists and helps older adults. This is also indicative of successful word-of-mouth propaganda via individuals who visited a LVCS and benefited from the helpful tips and tactics they learned about during their visits.

There are various specialists in the care network for older adults with vision problems. On the one hand, there are specialists in the medical field, the ophthalmologists and general practitioners, with some collaboration with specialists in other disciplines (e.g., ear, nose and throat medicine). Optometry can be viewed as situated somewhere between the medical and non-medical fields in healthcare, and older adults typically first contact the optician when their vision deteriorates or when a visual aid needs to be adjusted. On the other hand, there are the professionals in the non-medical domain– who primarily provide LVCS. This includes counsellors in the health system for the visually impaired and those in senior citizens’ organisations and other psychosocial counselling services (e.g., social counselling and psychological counselling centres). Therefore, the optometrist can be a vital gatekeeper in this network by linking older adults with the services of specific LVCS. In addition, an optometrist can provide critical support, address the initial social challenges and advise patients holistically. The World Council of Optometry (WCO) defines optometry as a “healthcare profession that is autonomous, educated, and regulated (licensed/registered), and optometrists are the primary healthcare practitioners of the eye and visual system who provide comprehensive eye and vision care, which includes refraction and dispensing, detection/diagnosis and management of disease in the eye, and the rehabilitation of conditions of the visual system” (13). In addition to delivering a comprehensive primary eye care service, optometrists have good communication skills and are easily accessible at high street practices with no wait times for appointments – and are well positioned as such. An improved utilisation of their services will enhance vision care for a population undergoing geriatric changes, with no need for additional infrastructure, i.e., at no additional cost to society (14).

### 4.1 Limitations

Given that this study focuses on Switzerland alone, the generalizability of our findings to contexts outside Switzerland may be limited. Nevertheless, our analysis of the data on Switzerland can serve as a valuable case study. Although the Swiss data allows us to examine referral practices, some important variables were not included in the survey. These variables include counselling content, success factors of a referral, and the personalities and social networks of older adults seeking to utilise LVCS. Furthermore, future studies using representative data should aim to investigate the factors that influence changes in referral practices and that may facilitate a highly comprehensive use of LVCS. This can be achieved with longitudinal studies that provide a deep understanding of referral practices over time. In addition, international data are necessary for insights into the state of referral practices outside Switzerland, including the role of optometrists in healthcare for older adults with VI.

## Data Availability

The raw data supporting the conclusions of this article will be made available by the authors, without undue reservation.
